# Geographic location determines beta‐cell autoimmunity among adult Ghanaians: Findings from the RODAM study

**DOI:** 10.1002/iid3.306

**Published:** 2020-05-07

**Authors:** Christiane S. Hampe, Diomira Sahabandu, Vivien Kaiser, Tanja Telieps, Liam Smeeth, Charles Agyemang, Joachim Spranger, Matthias B. Schulze, Frank P. Mockenhaupt, Ina Danquah, Olov Rolandsson

**Affiliations:** ^1^ Department of Medicine University of Washington Seattle Washington; ^2^ Institute of Tropical Medicine and International Health Charité–Universitaetsmedizin Berlin, Corporate Member of Freie Universitaet Berlin and Humboldt‐Universitaet zu Berlin, and Berlin Institute of Health Berlin Germany; ^3^ Department of Molecular Epidemiology German Institute of Human Nutrition Potsdam‐Rehbruecke Nuthetal Germany; ^4^ Helmholtz Center Munich Institute for Diabetes and Obesity Research Garching Germany; ^5^ Department of Non‐Communicable Disease Epidemiology London School of Hygiene and Tropical Medicine London UK; ^6^ Department of Public Health, Academic Medical Center, Amsterdam Public Health Research Institute University of Amsterdam Amsterdam The Netherlands; ^7^ Department of Endocrinology and Metabolism, DZHK (German Centre for Cardiovascular Research), Partner Site Berlin; Center for Cardiovascular Research (CCR), Charité–Universitaetsmedizin Berlin Corporate Member of Freie Universitaet Berlin and Humboldt‐Universitaet zu Berlin, and Berlin Institute of Health Berlin Germany; ^8^ Heidelberg Institute of Global Health Universitaetsklinikum Heidelberg Heidelberg Germany; ^9^ Department of Public Health and Clinical Medicine, Section of Family Medicine Umeå University Umeå Sweden

**Keywords:** diabetes mellitus, Ghana, glutamic acid decarboxylase autoantibodies, migration, zinc transporter‐8 autoantibodies

## Abstract

**Introduction:**

Beta‐cell autoantibodies are established markers of autoimmunity, which we compared between Ghanaian adults with or without diabetes, living in rural and urban Ghana and in three European cities.

**Methods:**

In the multicenter cross‐sectional Research on Obesity and Diabetes among African Migrants (RODAM) study (N = 5898), we quantified autoantibodies against glutamic acid decarboxylase (GAD65Ab) by radioligand binding assay (RBA) and established cut‐offs for positivity by displacement analysis. In a subsample, we performed RBA for zinc transporter‐8 autoantibodies (ZnT8Ab). Associations of environmental, sociodemographic, and clinical factors with GAD65Ab were calculated.

**Results:**

In this study population (age: 46.1 ± 11.9 years; female: 62%; Ghana‐rural: 1111; Ghana‐urban: 1455; Europe: 3332), 9.2% had diabetes with adult‐onset. GAD65Ab concentrations were the highest in Ghana‐rural (32.4; 10.8‐71.3 U/mL), followed by Ghana‐urban (26.0; 12.3‐49.1 U/mL) and Europe (11.9; 3.0‐22.8 U/mL) with no differences between European cities. These distributions were similar for ZnT8Ab. Current fever, history of fever, and higher concentrations of liver enzymes marginally explained site‐specific GAD65Ab concentrations. GAD65Ab positivity was as frequent in diabetes as in nondiabetes (5.4% vs 6.1%; *P* = .25). This was also true for ZnT8Ab positivity.

**Conclusion:**

Geographic location determines the occurrence of GAD65Ab and ZnT8Ab more than the diabetes status. Beta‐cell autoimmunity may not be feasible to differentiate diabetes subgroups in this population.

## Graphical abstract

Among 5898 Ghanaian adults living in rural Ghana, urban Ghana, and three European cities, GAD65Ab was strongly determined by the environment. Thus, GAD65Ab may not differentiate autoimmune subgroups of adult‐onset diabetes.

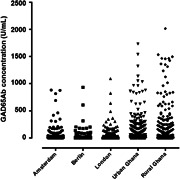

## 1 INTRODUCTION

Diabetes mellitus (henceforth, diabetes) constitutes a major challenge for international health. According to the World Health Organization (WHO), the number of adults with diabetes has doubled to 422 million between 1980 and 2014.^1^ While this rapid increase has largely been attributed to the emergence of type 2 diabetes in low‐ and middle‐income countries, the distinction between the major diabetes subtypes is not trivial. This might be particularly true for Africans in their country of origin and for African migrants in Europe; both groups are disproportionately affected by diabetes.^2^ Conventionally, the disease etiology determines diabetes subclassification and disease management.^3^ Type 1 diabetes is characterized by beta‐cell destruction and autoimmunity, and type 2 diabetes results from insulin resistance and/or insulin secretory defects.^3^ However, there is an overlap between these subtypes. In many non‐African populations, 5% to 10% of persons classified as type 2 diabetes are positive for autoantibodies directed against the beta‐cell autoantigen, glutamic acid decarboxylase 65 (GAD65Ab); this subgroup has been named latent autoimmune diabetes in adults (LADA).^4^ Moreover, even newer subtypes of adult‐onset diabetes have been proposed, including an adult autoimmune subtype.^5^ Thus, GAD65Ab are established sensitive and specific biomarkers of autoimmunity for newly diagnosed type 1 diabetes (T1D) at a young age and for adult‐onset autoimmune diabetes,^6^, ^7^, ^8^ including LADA.

The occurrence of GAD65Ab constitutes an essential decision criterion for diabetes management,^9^ predicts the rate of C‐peptide loss and the need for insulin treatment.^10^ Still, the utility of GAD65Ab for the identification of autoimmune diabetes for both, Africans in their home country and African migrants in Europe, has rarely been addressed. The few previous studies may not reflect the populations’ health care needs, because these were not population‐based samples.^11^, ^12^, ^13^

Therefore, we aimed at determining the population‐specific GAD65Ab prevalence as a marker for autoimmune diabetes among a representative sample of Ghanaian adults, and identifying the relationships with geographic location, sociodemographic and clinical factors.

## 2 MATERIALS AND METHODS

### 2.1 Study design and study population

The study protocol and procedures of the Research on Obesity and Diabetes among African Migrants (RODAM) study have been published elsewhere.^14^ In brief, this multicenter cross‐sectional study was conducted among Ghanaian adults (N = 6385; age range, 25‐70 years) in rural Ghana, urban Ghana, and Europe (Amsterdam, London, and Berlin) between July 2012 and September 2015. Response rates were 76% in rural Ghana, 74% in urban Ghana, 53% in Amsterdam, 75% in London, and 68% in Berlin.^14^

All blood samples were collected, handled, processed, and stored according to standardized procedures and were analyzed in the same laboratory in Berlin (Charité). In this cross‐sectional study, diabetes was defined as fasting plasma glucose (FPG) ≥ 7.0 mmol/L or documented glucose‐lowering medication or self‐reported diabetes. Medical history, lifestyle, and socioeconomic factors were recorded either by an ethnically matched staff in questionnaire‐based interviews or by self‐report.

### 2.2 Ethics statement

The RODAM study was conducted according to the guidelines laid down in the 1964 Declaration of Helsinki and its later amendments. All procedures involving human subjects were reviewed and approved by the respective ethics committees in Ghana, the Netherlands, the United Kingdom, and Germany. Written informed consent was obtained from all participants.

### 2.3 Questionnaire‐based interviews

Sociodemographic data were collected as previously described^14^ and included age, sex, length of stay in Europe, and educational level. Self‐reported medical history included age at diabetes diagnosis, family history of diabetes, type of diabetes medication (lifestyle, oral, insulin), insulin treatment immediately at diabetes diagnosis, insulin treatment within 6 months after diabetes diagnosis, current fever, and history of fever within the last 2 weeks. Smoking was categorized into current and former smokers or nonsmokers.

### 2.4 Anthropometric measurements

Anthropometric measures were taken in light clothing without shoes and to the nearest decimal. Weight was assessed with a person scale (SECA 877; SECA, Germany) and standing height with a portable stadiometer (SECA 217; SECA, Germany). Waist circumference was measured with a measuring tape. Body mass index (BMI) was calculated as weight (kg) divided by height (m^2^).

### 2.5 Biological sample processing and clinical biomarkers

Fasting venous blood samples were collected and aliquoted immediately after collection according to standard operating procedures and temporarily stored at −20°C. First early morning urine was collected. The separated blood samples and urine samples were stored at −80°C. FPG, C‐reactive protein (CRP), liver enzymes (aspartate aminotransferase [ASAT], alanine aminotransferase [ALAT], γ‐glutamyltransferase [GGT]), and biomarkers of kidney function (creatinine and urinary albumin) were measured using an ABX Pentra 400 Chemistry Analyzer (HORIBA ABX, Montpellier, France). For diabetes definition, we applied FPG because of the questionable utility of HbA1c in populations with frequent hemoglobinopathies and conditions that reduce erythrocyte lifespan. The estimated glomerular filtration rate (eGFR; mL/min/1.73 m^2^) was calculated using the abbreviated Modification of Diet in Renal Disease (MDRD) formula: 186 × (creatinine in mg/dL) − 1.154 × (age in years) − 0.203 × (0.742, if female) × (1.212, if African). Insulin concentrations were assessed using the Mercodia ELISA kit (Mercodia, Uppsala, Sweden) with a lower detection limit of 76 pmol/L. HbA1c (mmol/mol) was determined using the TOSOH G8 HPLC Analyzer (TOSOH Bioscience, Tokyo, Japan).

### 2.6 Measurements of beta‐cell autoantibodies

GAD65Ab was analyzed in all 5898 samples by radioligand binding assay (RBA) as previously described.^15^ Antibody levels were expressed as a relative index to correct for inter‐assay variation using the WHO standard for GAD65Ab.^16^ To determine the relative index, positive and negative control samples were included in all assays. The assay showed 70% sensitivity and 98% specificity for GAD65Ab in the International Combined Autoantibody Workshop.^17^ For statistical analyses and comparability, the index was translated into U/mL (Index 1 = 1000 U/mL).

To establish specific cut‐off values for GAD65Ab positivity in this population, we refrained from using arbitrary cut‐offs that are highly laboratory specific. Rather, a competition assay was performed in 59 participants in Ghana without diabetes and in 58 participants in Europe without diabetes, employing recombinant human (rh) GAD65 (Diamyd Medical, Sweden).^18^ The samples were incubated with [^35^S]‐GAD65 in the absence or presence of rhGAD65 (200 ng/mL). Samples whose binding to [^35^S]‐GAD65 were reduced by 40% in the presence of rhGAD65 were considered as having specific GAD65Ab (true positive) (Figure S1). This binding strength was achieved at higher GAD65Ab concentrations in samples from Ghana than in samples from Europe, and was set as the gold standard in subsequent receiver operating characteristic analysis (Figure S2). At 78% sensitivity and 70% specificity, the GAD65Ab cut‐offs were 121 and 97 U/mL for the Ghanaian and European study sites, respectively.

In subsamples, we assessed the robustness of GAD65Ab detection using alternative laboratory methods (Table S1). These methods used for GAD65Ab detection include (a) enzyme‐linked immunosorbent assay (ELISA) using a kit by Kronus (Boise) (n = 124), (b) ELISA using a kit by DRG International Inc (n = 84), and (c) luciferase immunoprecipitation system (LIPS) (n = 140) performed in an independent laboratory at Helmholtz Centre Munich, Germany.^19^ Also, we measured zinc transporter‐8 autoantibody (ZnT8Ab) as a novel marker for autoimmune diabetes. ZnT8Ab was measured under similar conditions as described for GAD65Ab with constructs containing the cytosolic segments (aa268‐369) encoding the aa325 codon variants, CGG (T) and TGG (W). Results for ZnT8Ab were converted into arbitrary units by extrapolation using a pan‐reactive positive serum from a patient with T1D with designated 1000 arbitrary units. Cut‐off was set at 10 U/mL for autoantibodies to ZnT8R and 18 U/mL for ZnT8W based on the 98th percentile observed in 162 individuals without diabetes.

Lastly, isotype‐specific identification of immunoglobulins (Ig) was performed using an ELISA (Bio‐Rad) in 316 samples (rural Ghana: n = 124; Europe: n = 192) to account for isotype shifts during different phases of the humoral immune response. Specifically, we measured total IgG 1, 2, 3, 4, IgE, IgA, and IgM. This analysis was performed because the RBA for GAD65Ab measurement does not discriminate GAD65Ab of different Ig isotypes.

### 2.7 Statistical analysis

Figure S3 shows the flow chart of imputed data, resulting in a final analytical sample size of 5898 participants. Multiple imputations were applied to estimate the missing values (n = 10; discriminant fully conditional specification method; relative efficiency: 97%‐99%). The characteristics of the study population are presented as mean ± standard deviation for normally distributed continuous variables, as median (interquartile range [IQR]) for non‐normally distributed continuous variables, and as the number of individuals (percentage) for categorical data. Comparisons across study sites and by diabetes status were made by Student's *t* test, the Wilcoxon rank‐sum test, and *χ* ^2^ test, respectively. Spearman correlations were used to investigate the relationships of GAD65Ab concentrations between different assays and with different isotypes. Next, we examined associations of place of residence, sociodemographic and clinical factors with GAD65Ab concentrations. Linear regression models were used to calculate adjusted means of GAD65Ab concentrations with their 95% confidence intervals. Study site and length of stay in Europe were included as important sociodemographic factors. Clinically relevant factors comprised duration of diabetes and insulin treatment. Particularly, we hypothesized that infectious agents may serve as potential environmental triggers for islet autoimmunity^11^, ^20^, ^21^ and explored the following factors: CRP, current fever, history of fever in the past 2 weeks, ALAT, ASAT, ASAT‐ALAT ratio, GGT, eGFR, and urinary albumin. The regression models were adjusted for age and sex (model 1), and additionally for education, BMI, waist circumference, and smoking status (model 2). In model 3, we further included infection‐related factors to assess whether they will mitigate the differences in GAD65Ab concentrations between study sites. By using a sensitivity analysis, we repeated the comparison by place of residence and diabetes status excluding individuals with self‐reported diabetes.

All analyses were performed using SAS 6.1 (SAS Institute Inc, Cary) and a 2‐sided *P* < .05 was considered statistically significant.

## 3 RESULTS

### 3.1 General characteristics

Table S2 displays the characteristics of the study population according to sex and place of residence. The mean age of the study population was 46.1 ± 11.9 years and the majority was female (62%). Individuals in Europe were mainly first‐generation migrants (98%) and their mean length of stay in Europe was 17.3 ± 8.8 years. Most participants had a lower or intermediate education (71%) and worked in manual jobs (68%). With regard to diabetes, the crude prevalence was 9.2% in the total study population, and the vast majority had an adult disease onset (median: 48 years; IQR: 40‐54 years). Diabetes was more common in men than in women (11% vs 8%), and more frequent in migrants than in nonmigrants (Europe: 11%, urban Ghana: 9%, rural Ghana: 5%). More than two‐thirds (69%) were reported to have diabetes, and 58% had an FPG ≥ 7 mmol/L. The median duration of diabetes was 5.0 years (IQR: 1.0‐11.0 years) and this was similar between study sites. Fifty‐four percent of the diabetes patients received medication: oral glucose‐lowering drugs (80%), lifestyle therapy (55%), and insulin treatment (15%).

### 3.2 GAD65Ab across study sites

**Figure 1:**
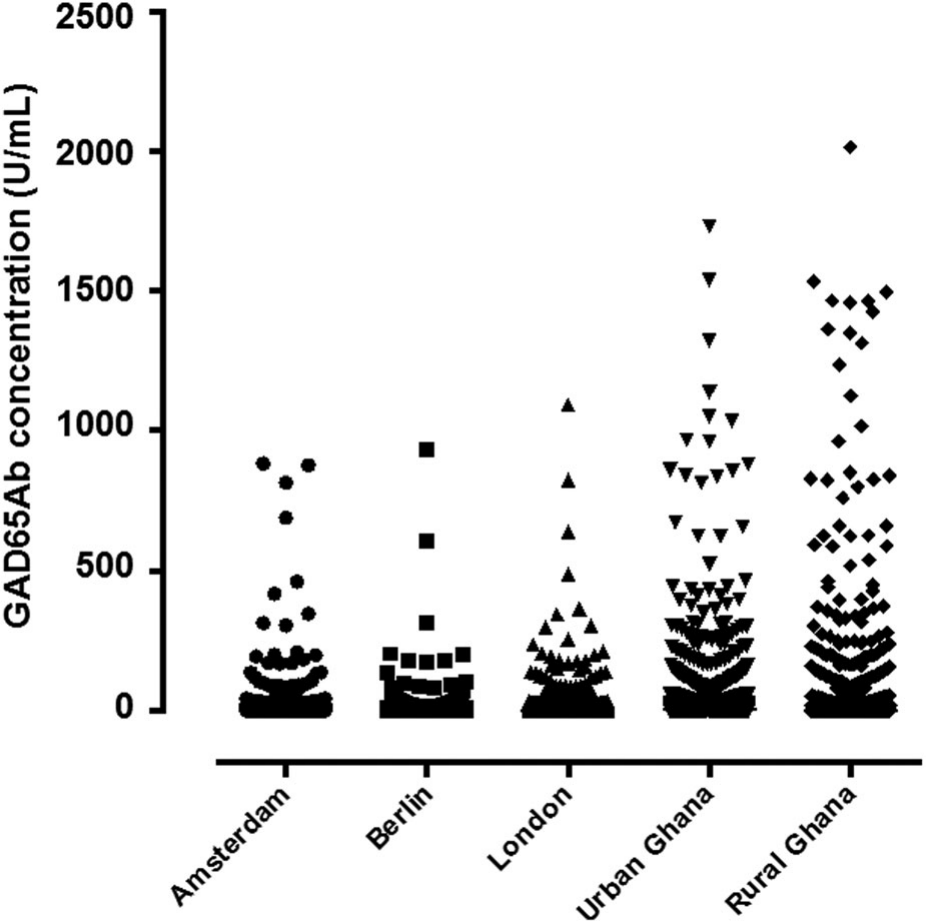
GAD65Ab concentrations (U/mL) in 5898 participants by study site

Figure 1 shows that individuals in rural Ghana had the highest GAD65Ab concentrations (median: 32.4; IQR: 10.8‐71.3 U/mL), followed by urban Ghana (median: 26.0; IQR: 12.3‐49.1 U/mL; *P* = .002) and Europe (median: 11.9; IQR: 3.0‐22.8 U/mL; *P* < .0001). No differences in GAD65Ab concentrations were seen between Amsterdam (median: 12.7; IQR: 4.2‐23.0 U/mL), London (median: 8.4; IQR: 0.3‐20.2 U/mL) or Berlin (median: 15.9; IQR: 7.4‐26.1 U/mL). Therefore, we combined the European study sites in the following analyses. Correspondingly, the proportions of individuals with GAD65Ab positivity was the highest in rural Ghana, followed by urban Ghana and Europe (Table 1).

**Table 1:** Proportions of GAD65Ab positivity by diabetes status and study site

	N	No diabetes (%)	Diabetes (%)	*P* value
Main analysis^a^				
Total	5898	6.1	5.4	.479
Rural Ghana	1111	13.9	14.3	.941
Urban Ghana	1455	8.3	8.9	.824
Europe	3332	2.4	2.6	.826
Sensitivity analysis^a^				
Total	5539	6.1	5.5	.728
Rural Ghana	1082	13.9	14.8	.896
Urban Ghana	1377	8.3	8.8	.907
Europe	3080	2.4	1.0	.380

^a^ For the main analysis, diabetes was defined as greater than or equal to 7 mmol/L or use of glucose‐lowering medication or self‐reported diabetes. For the sensitivity analysis, we excluded self‐reported diabetes.

The results obtained by the Kronus ELISA in 124 samples were similar to the RBA‐based measurements: the median GAD65Ab concentrations for Ghana and for Europe were 64.5 U/mL (IQR: 54.9‐101.8 U/mL) and 58.5 U/mL (IQR: 53.2‐68.9 U/mL; *P* = .069), respectively.

**Figure 2:**
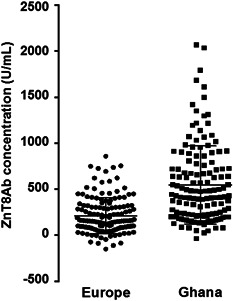
ZnT8Ab concentrations (U/mL) in 146 participants from Europe and 141 participants from Ghana. Median binding and standard deviations are indicated

The Pearson correlation between the Kronus ELISA and the RBA was *r* = .49 with *P* < .0001. For the DRG ELISA and the LIPS analysis, we cannot comment on site‐specific results, because these assays were only performed in samples from rural Ghana and Berlin, respectively (Table S1). Yet, as shown in Figure 2, ZnT8Ab concentrations were also significantly higher in Ghana than in Europe (*P* < .0001). ZnT8Ab correlated moderately with GAD65Ab concentrations (*r* = .24; *P* < .0001).

### 3.3 Immunoglobulin isotypes and IgG subclasses

The additional isotype analyses revealed that the most prevalent serum autoantibody isotype in all study sites was IgG, with IgG1 being the major subclass (Figure S4). IgG1 concentrations in samples from Ghana (median 13.4; IQR: 10.9‐16.7 mg/mL) were significantly higher compared with those from Europe (median: 11.7; IQR: 9.5‐13.6 mg/mL; *P* < .0001 and Figure S5). This was also true for IgM (0.56 vs 0.38 mg/mL; *P* < .0001) and IgE concentrations (0.20 vs 0.15 mg/mL; *P* < .0001; Figure S5). The correlations for GAD65Ab concentrations with Ig isotypes and IgG subclasses are presented in Figure S6. IgM, IgG, and IgE levels correlated with GAD65Ab concentrations in the 316 samples analyzed for all four parameters. However, when stratified by study site, only IgM levels correlated with GAD65Ab concentrations and only in Ghana (*r* = .28; *P* = .002).

### 3.4 Factors related to GAD65Ab concentrations

Taken together, GAD65Ab, IgG1, IgE, and IgM concentrations were significantly higher in Ghana than in Europe. These observations and the fact that other markers of infection were the highest in rural Ghana (Table S2) gave rise to the hypothesis that GAD65Ab might be related to an infectious environment. In Table 2, we present the means of GAD65Ab concentrations, adjusted for important sociodemographic and clinical factors. Residence in rural Ghana remained associated with higher GAD65Ab concentrations, followed by urban Ghana and Europe. For infection‐related factors, GAD65Ab concentrations were higher in individuals who had fever or reported a history of fever within the past 2 weeks. Also, higher concentrations of ASAT were associated with higher GAD65Ab concentrations. Further, the concentrations of CRP, ALAT, and ASAT tended to be directly associated with GAD65Ab concentrations (Table 2). No other clinically relevant factors, such as insulin treatment and the duration of diabetes, were associated with GAD65Ab concentrations. The differences in GAD65Ab concentrations between study sites remained when infection‐related factors were added as potential explanatory variables to sociodemographic and anthropometric factors.

**Table 2:** Adjusted means for GAD65Ab concentrations (U/mL) according to sociodemographic and clinical factors

Infection‐related factor	N	Adjusted means in U/mL (95% CI): model 1	Adjusted means in U/mL (95% CI): model 2	Adjusted means in U/mL (95% CI): model 3
Study site	5898			
Rural Ghana	1111	85.0 (78.3, 91.7)*	85.4 (76.5, 94.3)*	84.0 (74.9, 93.1)*
Urban Ghana	1455	58.2 (52.3, 64.2)	61.4 (53.9, 68.9)	60.7 (53.1, 68.3)
Europe	3332	22.0 (18.2, 25.9)	26.8 (21.3, 32.3)	27.3 (21.7, 32.8)
Length of stay in Europe, y	3332			
≤10	708	22.3 (18.2, 26.5)	22.8 (17.9, 27.7)	
11‐20	1431	21.7 (18.9, 24.6)	22.4 (18.6, 26.1)	
21‐30	973	19.9 (16.4, 23.5)	20.5 (16.4, 24.6)	
>30	220	25.0 (17.4, 32.5)	25.4 (17.6, 33.2)	
Duration of diabetes, y	541			
<2	142	31.2 (12.7, 49.7)	28.1 (6.8, 49.3)	
2‐5	114	30.9 (10.6, 51.1)	26.1 (3.3, 49.4)	
5‐10	118	30.9 (10.9, 50.9)	26.4 (3.8, 49.0)	
10‐20	115	40.0 (19.6, 60.4)	34.7 (11.7, 57.7)	
≥20	52	55.6 (25.8, 85.8)	50.5 (18.3, 82.8)	
Insulin treatment	541			
No	459	34.0 (23.8, 44.2)	38.7 (13.1, 64.2)	
Yes	82	42.1 (18.1, 66.0)	29.0 (14.0, 44.0)	
C‐reactive protein, mg/L	5898			
<3.0	1680	45.8 (40.2, 51.4)	39.7 (32.6, 46.7)	
3.0‐4.9	3385	40.3 (36.3, 44.3)	41.1 (35.4, 46.8)	
5.0‐9.9	488	44.8 (34.4, 55.2)	49.9 (38.7, 61.1)	
≥10.0	345	43.6 (21.3, 56.0)	46.4 (33.5, 59.4)	
Current fever	5898			
No	5611	41.2 (38.1, 44.3)*	40.9 (35.9, 46.1)*	
Yes	287	68.2 (54.7, 81.7)	61.5 (47.3, 75.6)	
Hx fever in the past 2 wk	5898			
No	4997	39.7 (36.4, 42.9)*	40.1 (34.9, 45.3)*	
Yes	901	58.9 (51.2, 66.6)	52.8 (43.9, 61.6)	
ALAT, U/L	5898			
Tertile 1 (≤16.3)	1977	40.9 (35.5, 46.3)	37.9 (31.2, 44.7)	
Tertile 2 (16.4‐23.0)	1953	43.9 (38.7, 49.2)	43.4 (36.7, 50.0)	
Tertile 3 (≥23.1)	1969	42.5 (37.3, 47.6)	43.5 (37.0, 50.1)	
ASAT, U/L	5898			
Tertile 1 (≤27.6)	1976	29.8 (24.1, 34.9)*	32.1 (25.5, 38.7)*	
Tertile 2 (27.7‐36.1)	1960	42.0 (36.8, 47.2)	41.2 (34.5, 47.8)	
Tertile 3 (≥36.2)	1962	54.4 (49.3, 59.5)	51.9 (45.3, 58.3)	
ASAT/ALAT	5898			
≤1.0	434	28.4 (14.5, 39.3)*	33.6 (21.9, 45.4)	
>1.0	5464	43.7 (40.5, 46.9)	42.4 (37.2, 47.5)	
GGT, U/L	5898			
Tertile 1 (≤25.3)	1971	42.3 (36.9, 47.7)	39.3 (32.5, 46.1)	
Tertile 2 (25.4‐37.5)	1965	47.0 (41.8, 52.2)	46.3 (39.6, 53.0)	
Tertile 3 (≥37.6)	1962	38.4 (33.3, 43.6)	39.9 (33.5, 46.4)	
MDRD‐eGFR, mL/min/1.73 m^2^	5898			
Tertile 1 (≤0.48)	1967	42.3 (36.9, 47.7)	43.0 (36.3, 49.8)	
Tertile 2 (0.49‐0.57)	1965	40.0 (34.8, 45.2)	39.8 (33.3, 46.3)	
Tertile 3 (≥0.58)	1966	45.1 (39.8, 50.4)	42.3 (35.6, 49.1)	
Urinary albumin, mg/L	5898			
0‐19.9	5014	42.5 (39.2, 45.8)	41.3 (36.1, 46.6)	
20.0‐29.9	277	47.9 (34.2, 61.6)	49.7 (35.6, 63.9)	
≥30.0	607	39.5 (30.3, 48.8)	40.9 (30.9, 51.0)	

*Note*: Model 1: linear regression adjusted for age and sex; model 2: linear regression adjusted for age, sex, educational level, smoking status, body mass index, and waist circumference; model 3: linear regression adjusted for all factors in model 2 and markers of infection.

Abbreviations: ALAT, alanine aminotransferase; ASAT, aspartate aminotransferase; CI, confidence interval; eGFR, estimated glomerular filtration rate; GGT, γ‐glutamyltransferase; MDRD, Modification of Diet in Renal Disease.

* *P *< .05 for trend.

### 3.5 GAD65Ab by diabetes status

**Figure 3:**
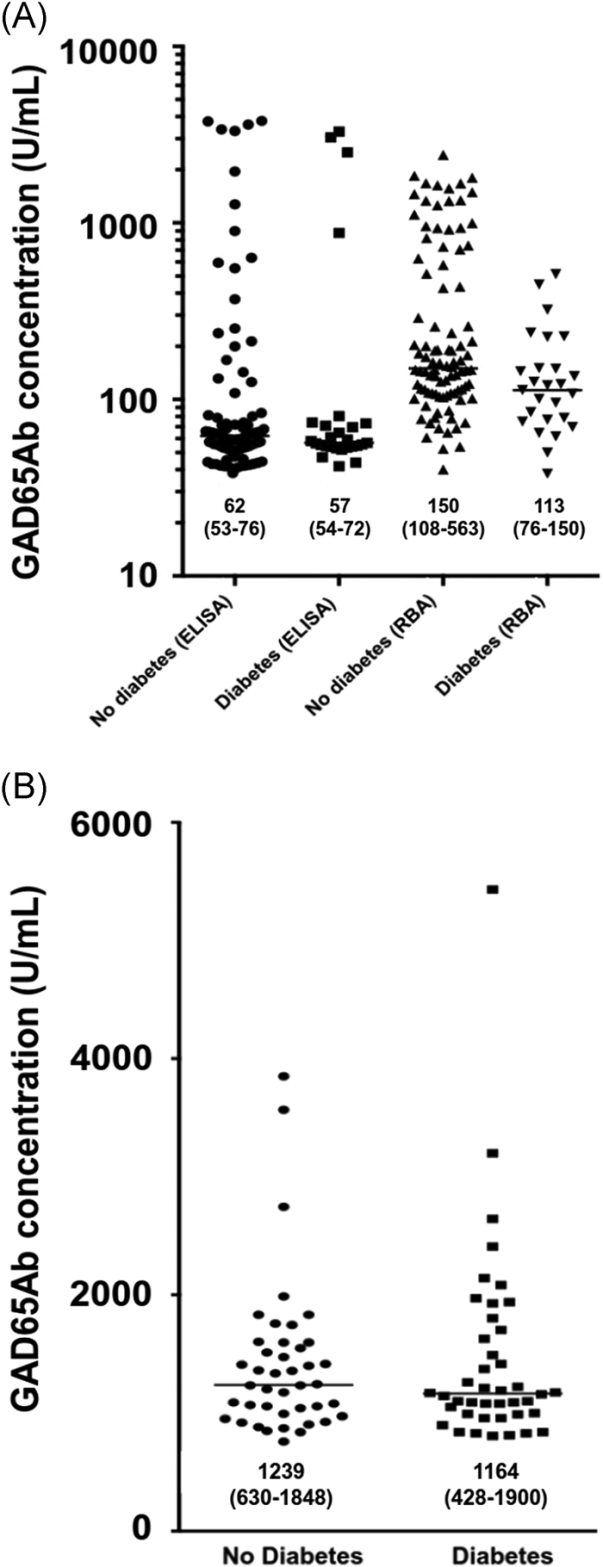
GAD65Ab concentrations (U/mL) using the radioligand binding assay (RBA) and two enzyme‐linked immunosorbent assays (ELISA). GAD65Ab titers (U/mL) obtained by Kronus ELISA in 124 samples (A, left), by RBA in 124 samples (A, right), and by DRG ELISA in 84 samples (B). Numbers indicate median (percentile 25‐percentile 75)

Table 1 also presents the proportions of GAD65Ab‐positive individuals according to diabetes status. In the present study population, 5.4% of the individuals in the group with adult‐onset diabetes were GAD65Ab‐positive, while the proportion of GAD65Ab positivity was 6.1% in the nondiabetes group (*P* = .48). In addition, there were no differences per study site in GAD65Ab positivity between diabetes and nondiabetes groups. These findings were confirmed when excluding participants with self‐reported diabetes (Table 1, bottom panel). The GAD65Ab concentrations by diabetes status obtained by the Kronus ELISA and by the DRG ELISA are shown in Figure 3, and were similarly distributed between individuals with or without diabetes. Also, the LIPS analysis showed similar concentrations of GAD65Ab for individuals with and without diabetes (median: 1.2 U/mL; IQR: 0.0‐4.8 U/mL vs median: 0.9 U/mL; IQR: 0.0‐4.0 U/mL; *P* = .62). This translated into 11% (8 out of 70) GAD65Ab‐positive individuals in the diabetes group and 10% (7 out of 70) GAD65Ab‐positive participants in the nondiabetes group (*P* = .79). For Ig isotypes and IgG subclasses, there were no differences between individuals with or without diabetes. Lastly, none of the individuals with diabetes‐onset before the age of 18 years (n = 11) were GAD65Ab‐positive.

## 4 DISCUSSION

### 4.1 Summary of main results

The present study aimed at determining the potential of beta‐cell autoantibodies to classify diabetes and to guide its therapy among 5898 Ghanaian adults. Using a highly specific detection method for GAD65Ab, surprisingly, we observed a similar prevalence of GAD65Ab positivity between individuals with and without adult‐onset diabetes. There was a geographic trend in GAD65Ab concentration with the highest concentrations in rural Ghana, followed by urban Ghana and Europe. However, this was only partly explained by markers of infection.

### 4.2 Strengths and limitations

The present study provides first‐time findings from a large sub‐Saharan African sample regarding biomarkers of adult‐onset autoimmune diabetes that have been established in mainly European populations. Nevertheless, our results need to be interpreted with caution. Participation in the study was limited to adults aged 25 to 70 years. Formerly, individuals with T1D in sub‐Saharan Africa had a poor prognosis.^22^ Given that greater than 98% of our middle‐aged study participants were born in Ghana, only a few individuals with T1D might have reached the age of inclusion. This survival effect could have led to an underrepresentation of T1D in our study population, which is further corroborated by the fact that most of the patients with diabetes in the present study had an adult age of disease onset. Hence, such survival effect might, in part, explain the similar prevalence of GAD65Ab positivity in diabetes and nondiabetes groups. While genetic information for the human leukocyte antigen region influence GAD65Ab concentrations, these data were not available for our study population. An important strength of our study is the comprehensive set of analyses to test the robustness of our results. We confirmed the specificity of GAD65Ab measurement by competition assays yielding 78% sensitivity and 70% specificity, and by the identification of Ig isotypes and IgG subclasses. We verified the differential proportions of GAD65Ab positivity according to diabetes status by two different ELISAs, by LIPS analysis in an independent laboratory, and after excluding individuals with self‐reported diabetes. Further, we had a range of objectively measured and self‐reported infection‐related factors at hand to identify their relationships with GAD65Ab concentrations.

### 4.3 GAD65Ab concentrations, ethnicity, and diabetes status

Previous reports indicate that the occurrence of GAD65Ab and their association with diabetes is ethnicity specific. The prevalence of GAD65Ab positivity in individuals without diabetes spans from 1% in Greenland Inuit through 2% to 3% in individuals with European descent up to 1% to 7% in non‐Hispanic blacks.^23^, ^24^, ^25^, ^26^ While Caucasian and Asian populations report 80% to 90% autoantibody positivity in T1D patients,^27^, ^28^ only 36% to 44% of T1D patients living in sub‐Saharan Africa tested positive for islet cell autoantibodies^13^, ^29^ and much lower frequencies (7%‐9%) were observed in T1D patients residing in Nigeria.^30^ This disconnect between T1D and autoantibodies was also observed in African Americans diagnosed with T1D,^31^ suggesting a strong ethnical factor in the development of beta‐cell autoantibodies. The association between GAD65Ab and diabetes can also be observed in patients diagnosed with adult‐onset diabetes in Europe, where the respective proportions reach differences of 1 to 4 percentage points.^25^, ^26^ However, this is not uniformly seen for individuals from sub‐Saharan Africa.^11^, ^23^ For African Americans in the Third National Health and Nutrition Examination Survey, there was only a trend for differences in GAD65Ab positivity between diabetes and nondiabetes groups (3.7% vs 1.3%; *P* = .08), whereas the figures for non‐Hispanic whites were clearly distinct: 6.3% vs 2.0%; *P* = .001.^23^ However, a small study from Kumasi, Ghana (n = 120) reported that 18% of patients with insulin‐dependent diabetes, 9% of patients with non‐insulin–requiring diabetes, and 2% of individuals without diabetes were positive for GAD65Ab.^11^ In that study, the patients were adults with new‐onset diabetes (mean age: 48.2 ± 13.4 years; disease duration <1 year), and GAD65Ab was measured by an ELISA developed by DRB International Inc. Notably, this assay uses anti‐human IgG for the detection of the GAD65‐human antibody complexes. The RBA in the present study and the KRONUS ELISA kit cannot discriminate between different Ig isotypes. It was, therefore, possible that the highly reactive IgM antibody interfered with the specific detection of GAD65 antibodies of the IgG isotype in the RBA. This is supported by our finding that IgM concentrations correlated with GAD65Ab. However, our analysis in a subset of diabetes and nondiabetes groups using the DRB ELISA kit showed no significant differences for GAD65Ab concentration between the two groups. Moreover, the results obtained by the DRB ELISA correlated significantly with the GAD65Ab measured by the RBA, suggesting that a substantial proportion of GAD65Ab are of the IgG isotype. Therefore, the discrepancies between the earlier study in Kumasi and ours might stem from differential population characteristics and sample size constraints in the study by Agyei‐Frempong et al.^11^ Taken together, our data suggest that GAD65Ab may not differentiate between autoimmune and nonautoimmune diabetes in specific ethnic subgroups.

### 4.4 Infectious environment and GAD65Ab

The different distributions of GAD65Ab across study sites were striking, and we hypothesized that exposure to infectious environments could contribute to this observation. IgM concentrations were the highest in rural Ghana, and only there, IgM moderately correlated positively with GAD65Ab. High IgM concentrations characterize acute and recurrent infections.^32^ In sub‐Saharan Africa, infectious diseases, such as malaria, still constitute a major public health challenge.^33^ Malaria is typically more prevalent in rural settings, and the humoral response against the malaria parasite *Plasmodium falciparum* is dominated by high IgG and IgM serum levels.^34^ Surprisingly, only a fraction of these antibodies are specific to malaria antigens, while the majority is polyclonal, showing reactivity to rheumatoid factor and antinuclear specificity.^35^ Importantly, the antibody concentrations remain high even after the curation of clinical malaria but are inversely correlated with years of residence in endemic areas.^35^, ^36^ Moreover, infections with parasites, such as *Plasmodium* sp., are known to initiate autoimmune responses in previously nonautoimmune individuals.^37^ The effect of an infectious environment on the production of autoantibodies was elegantly illustrated by the prevalence of antinuclear autoantibodies (ANA) among migrants from Nigeria and Ghana to Italy.^20^ These individuals showed significantly higher ANA concentrations than the Italian reference population, but the prevalence strongly decreased after longer length of residence in Italy (≥8 years). This was not as clearly seen in our study population, where 88% of individuals had been living in Europe for more than 10 years. Also, the different GAD65Ab concentrations between Ghana and Europe remained discernible even after inclusion of infection‐related factors in the final regression model. In fact, the low proportions of GAD65Ab positivity in the European sites of the present study population resemble those observed in the European Prospective Investigation into Cancer and Nutrition (EPIC)‐InterAct study in the same countries.^26^ Therefore, we speculate that alternative environmental factors such as higher exposure to air pollution, vaccines, family environment, and stress in Ghana as compared with Europe might be involved.^38^ Therefore, GAD65Ab does not serve as a specific marker for autoimmune diabetes in this sub‐Saharan African population.

## 5 CONCLUSIONS

Pending verification in independent sub‐Saharan African populations, our findings may have important implications for clinical management and health care planning for these population groups. Our results highlight the need to validate established markers for autoimmune diabetes in different ethnic populations and to develop new ones. Until then, for the growing group of migrants from sub‐Saharan Africa to Europe, other factors than autoimmune status may be more relevant for efficient and effective disease management and for the identification of novel diabetes subgroups.

## CONFLICT OF INTERESTS

The authors declare that there are no conflict of interests.

## Supporting information

Supporting information

Supporting information

Supporting information

## Data Availability

Restrictions apply to the availability of data analyzed during this study to preserve participant confidentiality. The corresponding author will on request detail the restrictions and any conditions under which access to some data may be provided.
