# Supplementary material for: Geographic location determines beta‐cell autoimmunity among adult Ghanaians: Findings from the RODAM study
Source: Immun Inflamm Dis. 2020 May 7;8(3):299–309. doi: 10.1002/iid3.306 (PMC7416037; doi:10.1002/iid3.306)

Figure S1. Competition assays of GAD65Ab in Ghana and Europe

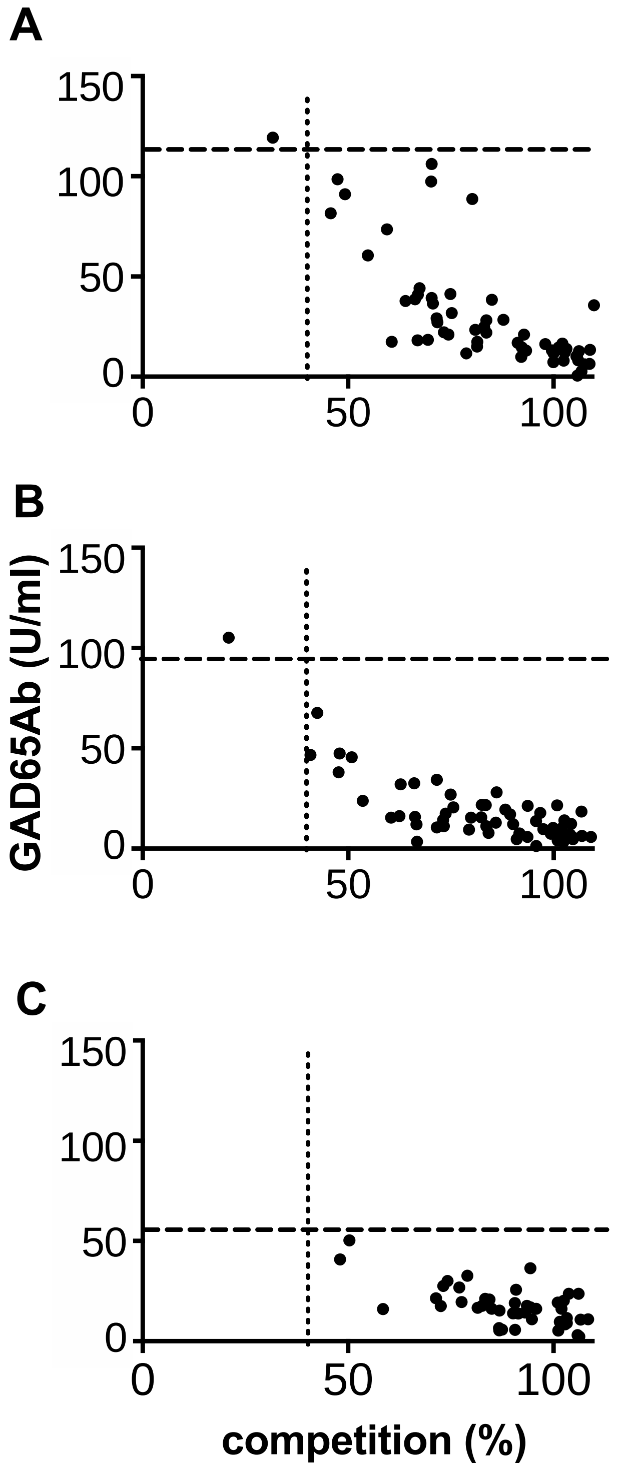

Binding of serum samples to radiolabeled GAD65: **(A)** 59 Ghanaian adults without diabetes, **(B)** 58 Ghanaian migrants in Europe without diabetes, and **(C)** 44 European adults in Seattle without diabetes. Vertical lines indicate the cut-off for the specificity of binding, while horizontal lines indicate the corresponding cut-off to define GAD65Ab positivity.

Figure S2. Receiver Operating Characteristic (ROC) analyses for Ghana and Europe

A

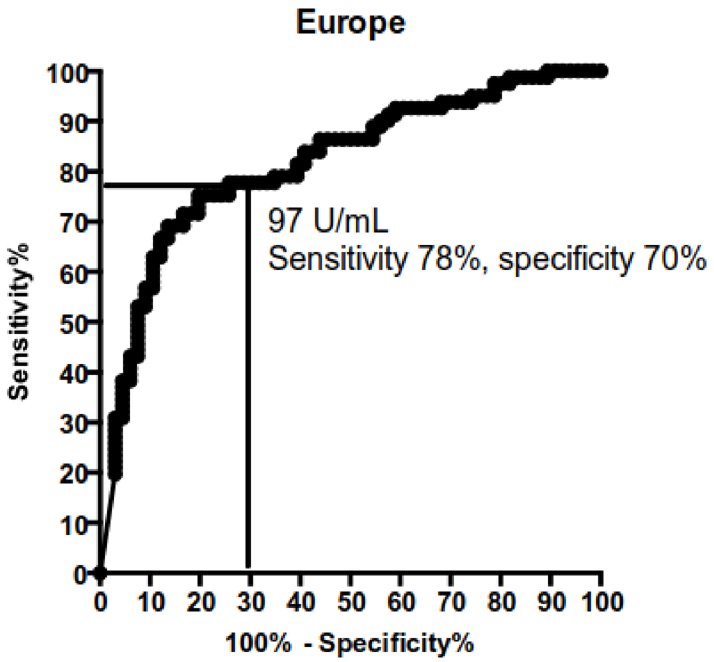

B

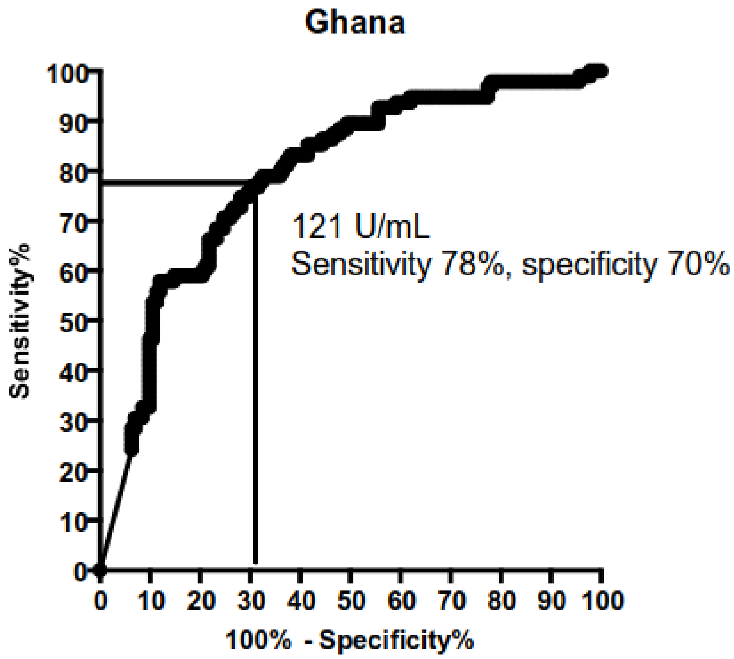

Receiver Operating Characteristic (ROC) analyses for reduced binding strength to ^{35}S-GAD65 in the presence of 40% recombinant human (rh)GAD65 during competition assay (true positive); **(A)** 59 Ghanaian adults without diabetes; **(B)** 58 Ghanaian migrants in Europe without diabetes

Figure S3. Flow chart of excluded participants and imputed data

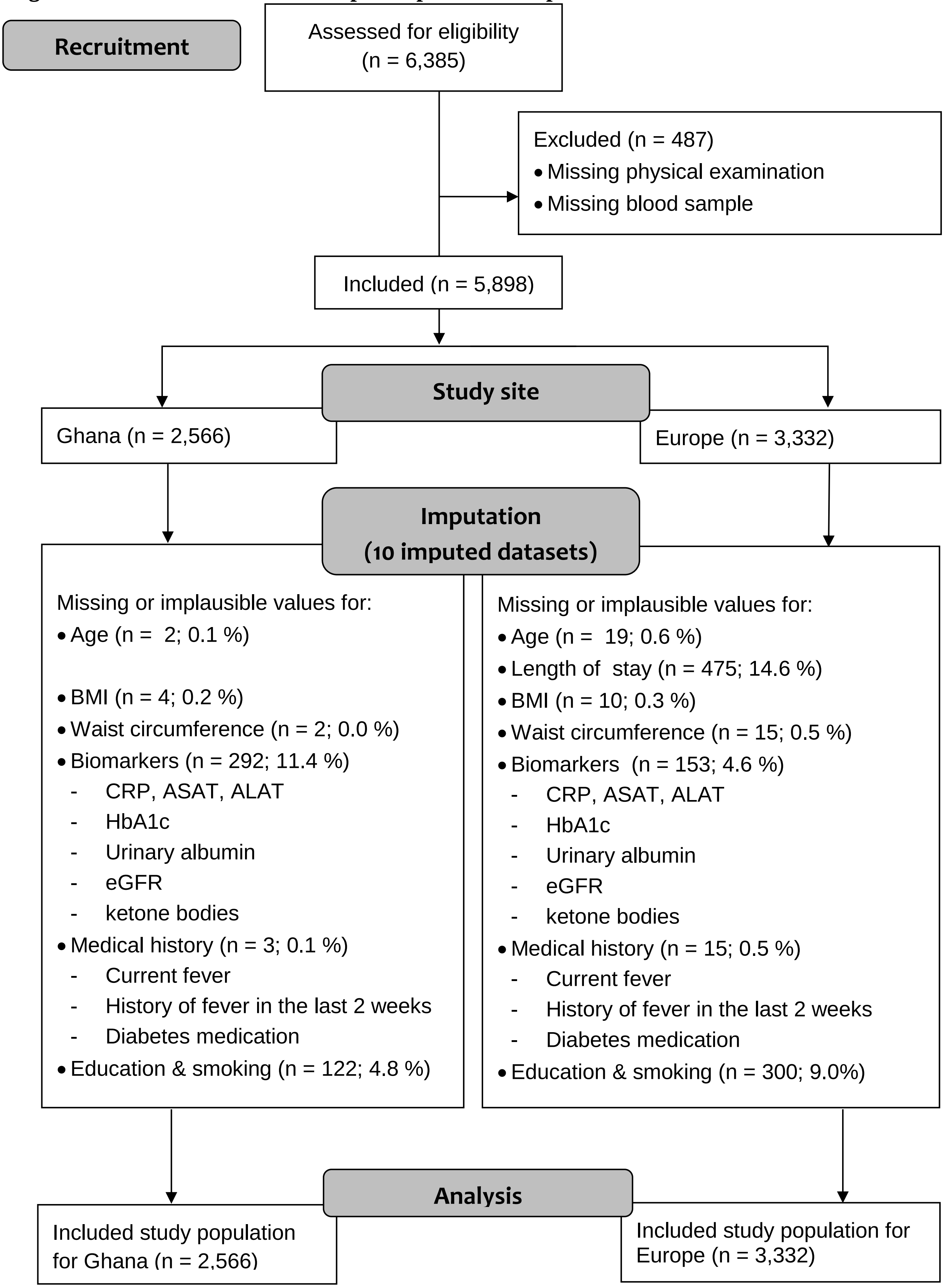

Figure S4. Immunglobulin (Ig) isotypes and IgG subclasses (mg/mL) in 316 participants by study site

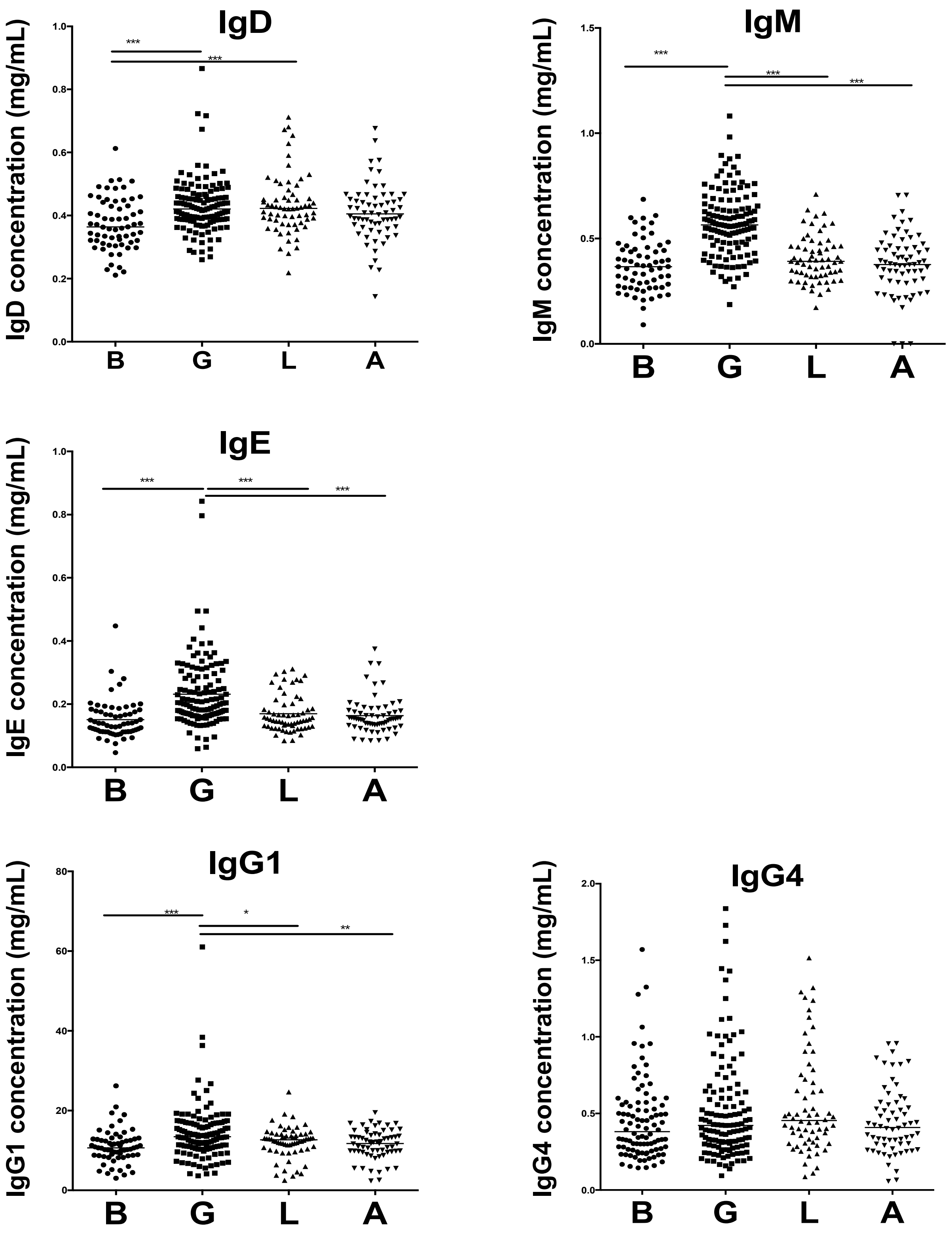

Immunoglobulin isotypes IgD, IgM, IgE and IgG subclasses IgG1, IgG2, IgG3, and IgG4 are shown in mg/mL: B, Berlin; G, Ghana; L, London; A, Amsterdam. Median concentrations are indicated. Significant differences in immunoglobulin concentrations between study sites are indicated by horizontal bars and asterisks (*, p-value <0.05-0.01; **, p-value <0.01-0.001; ***, p-value <0.0001).

Supplemental Figure S5. Immunoglobulin (Ig) isotopes and IgG subclasses (mg/mL) in 316 participants

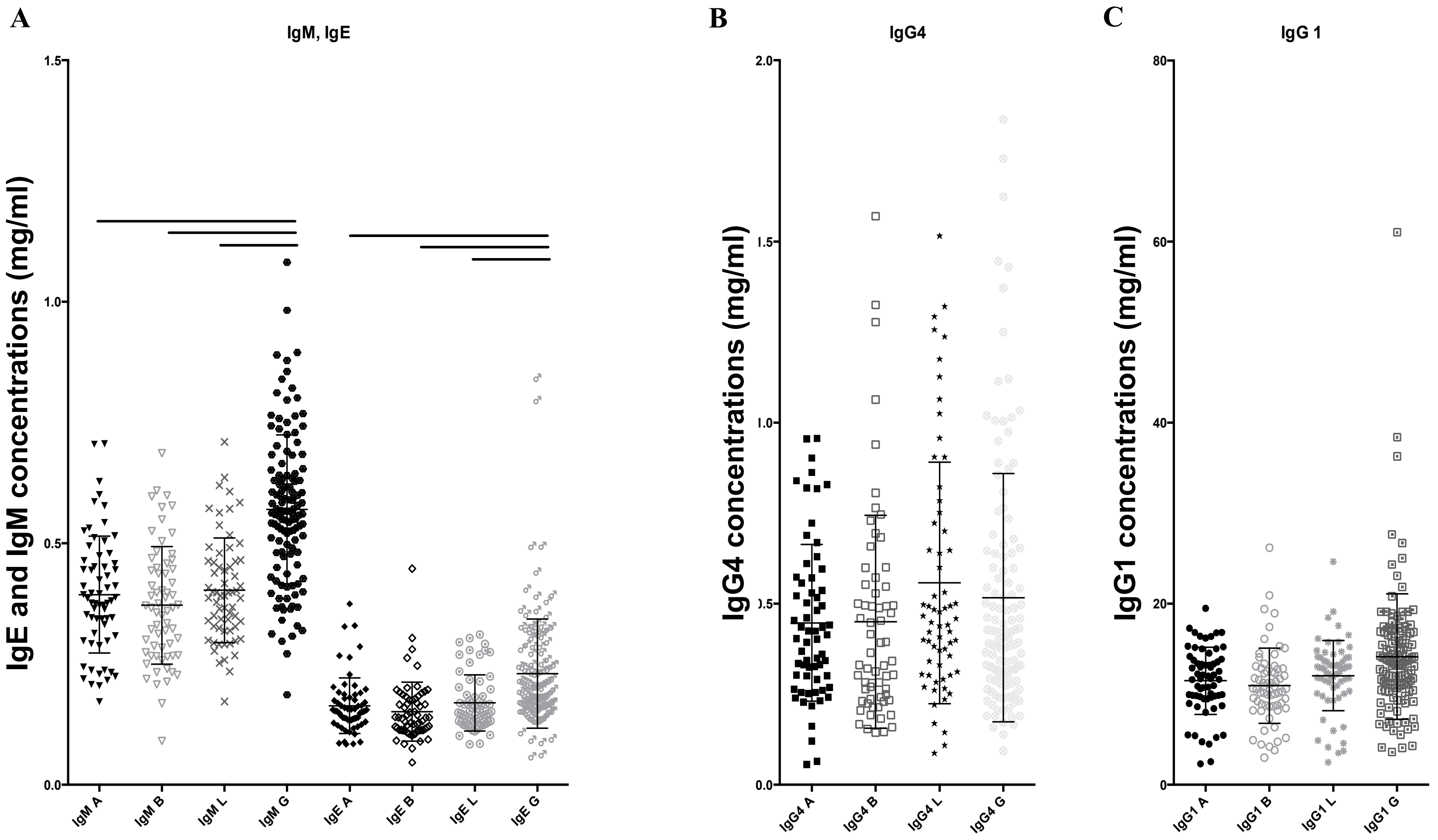

Subclasses of IgM and IgE (A), IgG4 (B), and IgG1 (C) are shown for 316 samples (124 rural Ghana, 64 London, 64 Berlin and 64 Amsterdam). Median concentrations are indicated. Significant differences in immunoglobulin concentrations between study sites are indicated by horizontal bars and asterisks (p-value <0.05-0.01: *, p-value <0.01-0.001: **, p-value <0.0001: ***).

Figure S6. Spearman correlations between GAD65Ab and immunoglobulin isotypes and IgG subclasses concentrations in 316 participants

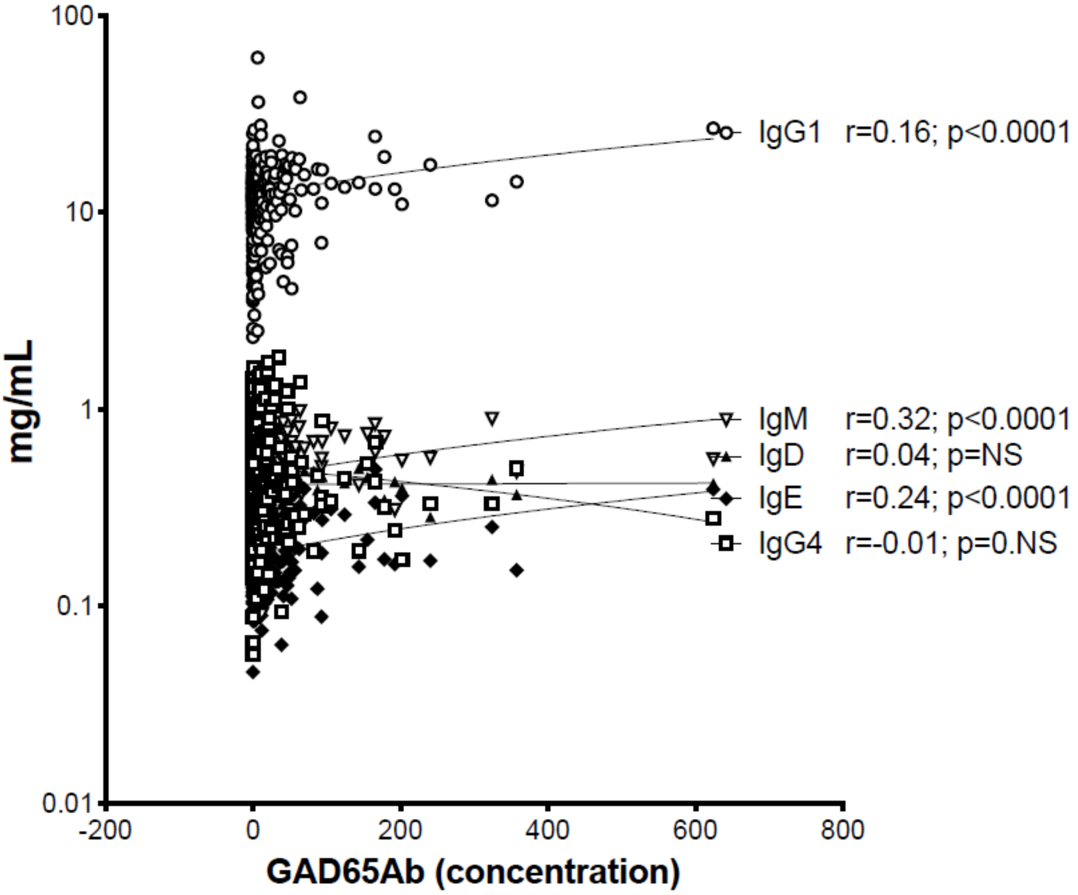

Supplement: Supplementary file 1 — Supporting information [file IID3-8-299-s001.pdf]
