# Supplementary material for: Geographic location determines beta‐cell autoimmunity among adult Ghanaians: Findings from the RODAM study
Source: Immun Inflamm Dis. 2020 May 7;8(3):299–309. doi: 10.1002/iid3.306 (PMC7416037; doi:10.1002/iid3.306)
Supplement: Supplementary file 2 — Supporting information [file IID3-8-299-s002.docx]

**Supplemental Table S1. Overview of beta-cell autoantibody assessments in the RODAM Study population**

| **Beta-cell autoantibody** | **N** | **Sample characteristics** | **Assay** | **Cut-off to define positivity** | **Intra-assay coefficient of variation** |
| --- | --- | --- | --- | --- | --- |
| GAD65Ab | 5,898 | Rural Ghana: 1,111  Urban Ghana: 1,455  Europe: 3,332 | Radioligand binding assay (RBA) | Ghana: >121 U/mL  Europe: >97 U/mL | 1-10% |
| GAD65Ab | 126 | Rural Ghana: 25  Urban Ghana: 31  Europe: 70 | Enzyme-linked immunosorbent assay (ELISA) by Kronus, Boise, USA | >5 U/mL | 3-16% |
| GAD65Ab | 84 | Rural Ghana: 84  (42 DM and 42 age-matched non-DM) | ELISA by DRG International, Inc., EIA, USA | >1.05 U/mL | 5% |
| GAD65Ab | 140 | Berlin: 140  (70 DM and 70 age- and sex-matched non-DM) | Luciferase immunoprecipitation system (LIPS) | ≥12.5 U/mL | 13% |
| ZnT8Ab | 287 | Rural Ghana: 141  Europe: 146 | Radioligand binding assay (RBA) | >10 U/ml for ZnT8R Ab >18 U/ml for ZnT8W Ab | 1-14% |

GAD65Ab, glutamic-acid decarboxylase 65 autoantibody; ZnT8Ab, zinc transporter-8 autoantibody

**Supplemental Table S2. General characteristics of the RODAM study population, by sex and study site (N = 5,898)**

| **Characteristics** | **Total**  **(n = 5,898)** | **Men**  **(n = 2,229)** | **Women**  **(n = 3,669)** | **Rural Ghana**  **(n = 1,111)** | **Urban Ghana**  **(n = 1,455)** | **Europe**  **(n = 3,332)** |
| --- | --- | --- | --- | --- | --- | --- |

| **Socio-demographic characteristics** |
| --- |

| Age (years) | 46.1 ± 11.9 | 46.8 ± 12.2 | 45.7 ± 11.7 | 48.4 ± 14.4 | 45.3 ± 11.5 | 45.7 ± 11.0 |
| --- | --- | --- | --- | --- | --- | --- |
| Sex (female, %) | 62.2 | - | - | 61.0 | 71.2 | 58.7 |
| Length of stay in Europe (years) | 17.3 ± 8.8 | 17.1 ± 9.1 | 17.3 ± 8.6 | - | - | 17.3 ± 8.8 |
| Migration generation (1st, %) | 98.4 | 98.7 | 98.2 | - | - | 97.1 |
| Education (%) |  |  |  |  |  |  |
| None/elementary | 34.2 | 20.8 | 42.3 | 58.2 | 43.5 | 22.1 |
| Lower | 36.5 | 40.0 | 34.5 | 30.6 | 39.1 | 37.4 |
| Intermediate | 18.3 | 22.6 | 15.7 | 7.4 | 12.5 | 24.4 |
| Higher | 11.0 | 16.7 | 7.6 | 3.8 | 43.9 | 16.1 |
| Occupation (%) |  |  |  |  |  |  |
| Non-manual | 31.9 | 30.2 | 32.9 | 12.7 | 35.9 | 36.5 |
| Manual | 68.1 | 69.8 | 67.1 | 87.3 | 64.1 | 63.5 |
| Smoking (ever, %) | 10.3 | 20.5 | 4.0 | 9.5 | 7.1 | 11.9 |
| **Clinical characteristics** | | | | | | |
| Diabetes* (yes, %) | 9.2 | 10.7 | 8.2 | 5.0 | 9.3 | 10.5 |
| Diabetes in family (yes, %) | 20.1 | 19.1 | 20.7 | 11.2 | 20.6 | 22.8 |
| Duration of diabetes (years) | 5.0 (1.0-11.0) | 5.0 (2.0-11.0) | 5.0 (1.0-11.0) | 4.5 (1.0-13.0) | 4.0 (1.0-9.0) | 5.0 (2.0-11.0) |
| Fasting glucose (mmol/L) | 5.4 ± 1.8 | 5.5 ± 2.0 | 5.3 ± 1.6 | 5.1 ± 1.6 | 5.6 ± 2.2 | 5.3 ± 1.6 |
| HbA1c (mmol/mol) | 38.3 ± 12.1 | 38.3 ± 12.6 | 38.1 ± 11.6 | 32.2 ± 9.7 | 38.8 ± 15.4 | 40.0 ± 10.2 |
| HbA1c (%) | 5.7 ± 1.1 | 5.7 ± 1.2 | 5.6 ± 1.1 | 5.1 ± 0.9 | 5.7 ± 1.4 | 5.8 ± 0.9 |
| GAD65Ab (U/mL) | 16.8 (5.5-35.4) | 18.4 (6.4-38.5) | 15.9 (4.8-33.4) | 32.4 (10.8-71.3) | 26.0 (12.3-49.1) | 11.9 (3.0-22.8) |
| GAD65Ab positivity (yes, %) | 6.1 | 7.1 | 5.4 | 14.0 | 8.4 | 2.4 |
| C-reactive protein (mg/L) | 0.7 (0.2-2.5) | 0.5 (0.2-1.5) | 1.0 (0.3-3.2) | 0.8 (0.2-2.9) | 0.8 (0.2-3.1) | 0.7 (0.2-2.2) |
| Current fever (yes, %) | 4.9 | 3.9 | 5.5 | 11.3 | 5.9 | 2.3 |
| History of fever, past 2 wks (yes, %) | 15.3 | 11.8 | 17.4 | 30.7 | 20.6 | 7.8 |
| ALAT (U/L) | 19.3 (14.9-25.7) | 22.9 (17.5-30.8) | 17.6 (13.9-22.6) | 18.7 (14.9-24.8) | 19.4 (15.0-25.9) | 19.4 (14.8-25.9) |
| ASAT (U/L) | 31.4 (25.7-39.0) | 34.4 (28.5-42.6) | 29.5 (24.4-36.7) | 35.9 (30.3-43.0) | 34.4 (28.7-41.6) | 28.6 (23.9-35.8) |
| ASAT/ALAT | 1.6 (1.3-2.0) | 1.5 (1.2-1.9) | 1.7 (1.4-2.0) | 1.9 (1.5-2.3) | 1.7 (1.4-2.1) | 1.5 (1.2-1.8) |
| GGT (U/L) | 30.6 (23.0-42.8) | 36.6 (26.8-51.8) | 27.8 (21.6-37.3) | 29.4 (22.3-42.3) | 31.5 (23.9-43.2) | 30.6 (22.9-42.8) |
| Serum creatinine (µmol/L) | 83.0 (71.7-95.7) | 96.4 (86.6-107.2) | 75.9 (67.9-85.0) | 81.3 (70.1-92.5) | 82.8 (71.5-95.2) | 83.7 (72.4-97.0) |
| eGFR (ml/min/1.73 m^2^) | 0.53 (0.46-0.61) | 0.54 (0.47-0.61) | 0.52 (0.46-0.61) | 0.54 (0.46-0.64) | 0.52 (0.45-0.59) | 0.53 (0.47-0.61) |
| Urinary albumin (mg/L) | 4.0 (4.0-11.8) | 4.0 (4.0-11.6) | 4.0 (4.0-11.9) | 4.0 (4.0-8.5) | 4.0 (4.0-14.3) | 4.0 (4.0-11.9) |
| Microalbuminuria (yes) | 15.0 | 14.9 | 15.0 | 10.2 | 18.5 | 15.1 |

| **Morphometric characteristics** |
| --- |

| Body mass index (kg/m^2^) | 27.1 ± 5.5 | 25.2 ± 4.5 | 28.3 ± 5.7 | 22.5 ± 4.2 | 26.9 ± 5.3 | 28.8 ± 5.0 |
| --- | --- | --- | --- | --- | --- | --- |
| Overweight (yes, %) | 35.5 | 37.2 | 34.4 | 17.7 | 34.2 | 42.0 |
| Obesity (yes, %) | 27.5 | 12.9 | 36.4 | 5.4 | 25.7 | 35.7 |
| Waist circumference (cm) | 90.4 ± 12.7 | 87.8 ± 12.1 | 91.9 ± 12.8 | 81.1 ± 10.7 | 89.3 ± 11.9 | 93.9 ± 12.0 |
| Abdominal obesity (yes, %) | 42.0 | 11.2 | 60.7 | 19.9 | 42.7 | 49.0 |

Data are presented as mean ± standard deviation for normally distributed continuous variables, as median (interquartile range) for skewed continuous variables, and as percentage for categorical variables. *, defined as fasting plasma glucose ≥7 mmol/L or use of glucose-lowering medication or self-reported diabetes
