# Supplementary material for: Geographic location determines beta‐cell autoimmunity among adult Ghanaians: Findings from the RODAM study
Source: Immun Inflamm Dis. 2020 May 7;8(3):299–309. doi: 10.1002/iid3.306 (PMC7416037; doi:10.1002/iid3.306)
Supplement: Supplementary file 3 — Supporting information [file IID3-8-299-s003.docx]

Figure S1: Competition assays of GAD65Ab in Ghana and Europe

Binding of serum samples to radiolabeled GAD65: (A) 59 Ghanaian adults without diabetes, (B) 58 Ghanaian migrants in Europe without diabetes, and **(C)** 44 European adults in Seattle without diabetes. Vertical lines indicate the cut-off for the specificity of binding, while horizontal lines indicate the corresponding cut-off to define GAD65Ab positivity.

Figure S2: Receiver Operating Characteristic (ROC) analyses for Ghana and Europe

ROC analysis for (A) 59 Ghanaian adults without diabetes and for (B) 58 Ghanaian migrants in Europe without diabetes

Figure S3:

Cut-offs for GAD65Ab positivity were calculated by Receiver Operator Characteristic (ROC) analysis using a competition assay as described in Figures S1 and S2. For the Ghanaian study sites, the cut-off was 121 U/mL, and for the European study sites, the cut-off was 97 U/mL (both with 78% sensitivity and 70% specificity).

Figure S4: Immunglobulin (Ig) isotypes and IgG subclasses (mg/mL) in 316 participants by study site

Immunoglobulin isotypes IgD, IgM, IgE and IgG subclasses IgG1, IgG2, IgG3, and IgG4 are shown in mg/mL: B, Berlin; G, Ghana; L, London; A, Amsterdam. Median concentrations are indicated. Significant differences in immunoglobulin concentrations between study sites are indicated by horizontal bars and asterisks (*, p-value <0.05-0.01; **, p-value <0.01-0.001; ***, p-value <0.0001).

Figure S5: Spearman correlations between GAD65Ab and immunoglobulin isotypes and IgG subclasses concentrations in 316 participants
